# Fabry disease in India: A multicenter study of the clinical and mutation spectrum in 54 patients

**DOI:** 10.1002/jmd2.12156

**Published:** 2020-08-15

**Authors:** Sheela Nampoothiri, Dhanya Yesodharan, Amrita Bhattacherjee, Hisham Ahamed, Ratna Dua Puri, Neerja Gupta, Madhulika Kabra, Prajnya Ranganath, Meenakshi Bhat, Shubha Phadke, Akella Radha Rama Devi, Sujatha Jagadeesh, Sumita Danda, Padmavathy Narayana Sylaja, Kausik Mandal, Sunita Bijarnia‐Mahay, Ravinder Makkar, Ishwar Chander Verma, Ashwin Dalal, Uma Ramaswami

**Affiliations:** ^1^ Department of Pediatric Genetics Amrita Institute of Medical Sciences and Research Centre Cochin Kerala India; ^2^ Diagnostics Division Centre for DNA Fingerprinting and Diagnostics (CDFD) Hyderabad India; ^3^ Department of Cardiology Amrita Institute of Medical Sciences and Research Centre Cochin Kerala India; ^4^ Institute of Genetics and Genomics Sir Ganga Ram Hospital New Delhi India; ^5^ Division of Genetics, Department of Pediatrics All India Institute of Medical Sciences New Delhi India; ^6^ Department of Medical Genetics Nizam's Institute of Medical Sciences Hyderabad India; ^7^ Department of Clinical Genetics Centre for Human Genetics Bangalore India; ^8^ Department of Medical Genetics Sanjay Gandhi Postgraduate Institute of Medical Sciences Lucknow India; ^9^ Rainbow Children Hospital Hyderabad India; ^10^ Department of Clinical Genetics and Genetic Counseling Mediscan Systems Chennai India; ^11^ Department of Clinical Genetics Christian Medical College and Hospital Vellore India; ^12^ Comprehensive Stroke Care Program, Sree Chitra Tirunal Institute for Medical Sciences and Technology (SCTIMST) Trivandrum Kerala India; ^13^ Sanofi Genzyme New Delhi India; ^14^ Lysosomal Disorders Unit, Institute of Immunity and Transplantation Royal Free London NHS Foundation Trust London UK

**Keywords:** chronic renal failure, Fabry disease, *GLA* mutation, hypertrophic cardiomyopathy, late onset, stroke

## Abstract

Fabry disease (FD) is a treatable X linked lysosomal storage disorder with a wide phenotypic spectrum. There is a scarcity of published data on the burden of FD in India. This study evaluates the clinical and molecular spectrum of Indian patients with FD. In this multicentric study involving 10 tertiary referral centers in India, we analyzed the clinical course and genotype of 54 patients from 37 families. Family screening identified 19 new patients (35%) from 12 index cases. Then, 33 *GLA* gene variants were identified in 49/54 (90.7%) which included 11 novel and 22 known pathogenic variants. Of the 54 patients in our cohort, 40 patients had “classical” and 10 patients had a “nonclassical” presentation. The symptoms and signs included kidney dysfunction in 38/54 (70.3%), neuropathic pain in 34/54 (62.9%), left ventricular hypertrophy in 22/49 (44.8%) and stroke in 5/54 (9.2%). Female heterozygotes were 10/54 (18.5%) of whom 2 were index cases. There was a significant delay in reaching the diagnosis of 11.7 years. Enzyme replacement therapy was initiated in 28/54 (51.8%) patients with significant improvement of neuropathic pain and gastrointestinal symptoms. This study highlights the clinical presentation and mutational spectrum of FD in India and suggests that family screening and screening of high‐risk groups (hypertrophic cardiomyopathy, idiopathic chronic renal failure and cryptogenic stroke) could be the most cost‐effective strategies for early identification of FD.

**Synposis**

Enzyme analysis for Fabry disease should be an integral part of the protocol for evaluation of patients with idiopathic chronic renal failure, hypertrophic cardiomyopathy and cryptogenic stroke.

## 1 INTRODUCTION

Fabry disease (FD, OMIM 301500) is an X linked lysosomal storage disorder caused by a mutation in *GLA* gene. Deficient or absent activity of enzyme α‐galactosidase A (α‐gal A) results in progressive accumulation of globotriaosylceramide (GL‐3, Gb3) and its deacylated form globotriaosylsphingosine (Lyso Gb3) within lysosomes of various cells leading to multiorgan involvement. *GLA* has seven exons and is located at Xq22. Nearly 700 *GLA* variants have been reported.^1^

The estimated prevalence of FD is 1/40 000 to 1/117 000 live births.^2^ It has a wide spectrum of clinical phenotypes ranging from the “classic” severe phenotype in males to totally asymptomatic females, with a group of patients in between with predominant cardiac or renal involvement presenting as “late onset” variants. Plasma Lyso‐Gb3 is a useful biomarker that differentiates classical from nonclassical FD in males. In patients with the classical onset phenotype, symptoms can manifest by 3 to 10 years in boys and around 13 years in girls.^2^ The spectrum of severity in heterozygous females ranges from asymptomatic to the classic severe phenotype probably due to skewed X‐chromosome inactivation.^2^, ^3^ Whereas demonstration of α‐gal A deficiency is a definitive method of diagnosis in affected males, it fails to identify up to 60% of affected females and therefore molecular studies of the *GLA* gene is the preferred diagnostic method in females.^4^ Dried blood spots are useful for enzymatic assay owing to the stability of enzymes for 6 months, but it is advisable to measure enzyme activity in leucocytes.^2^

Early diagnosis of FD is challenging. The average delay in diagnosis of FD is around 15 years.^2^ The life expectancy of untreated males and females is reduced by 20 years and 10 years respectively owing to progressive renal, cerebrovascular and cardiovascular complications.^2^

## 2 MATERIALS AND METHODS

### 2.1 Inclusion and exclusion criteria

Data were obtained from patients with FD from 10 major centers in India who presented between January 2007 and September 2019. Patients with a definitive diagnosis of FD (symptomatic or asymptomatic) confirmed by deficient levels of α‐gal activity and /or an identifiable pathogenic mutation in the *GLA gene* (hemizygous or heterozygous), were included in the study.

α‐gal A enzyme activity was performed on leucocytes or dried blood spots. Mutation analysis of *GLA* gene was performed using genomic DNA extracted from whole blood samples in accredited laboratories.

The variants identified were interpreted for pathogenic potential based on ACMG criteria and were characterized using protein modeling studies (details in Supplementary material).

## 3 RESULTS

**FIGURE 1:**
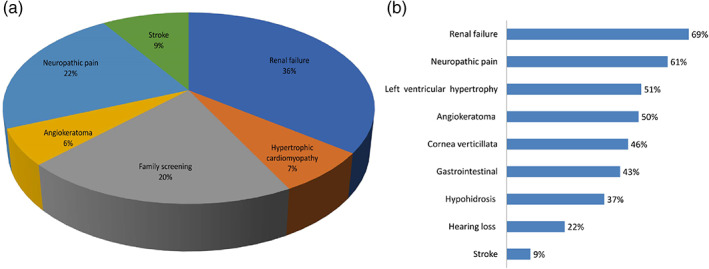
A, Symptoms at presentation in our cohort. B, Clinical signs in our cohort

Table 1 provides details of the 54 patients (44 males, 10 females) from 37 families with proven FD. Figure 1A,B details the symptoms and signs at presentation. Lyso Gb3 estimation was available in 12/54 patients and the mean value was 27.57 ng/mL (normal <1.8). Of the 54 patients in our cohort, only 10 patients had features consistent with the “nonclassic” phenotype. The mean age at onset of first symptom was 14 ± 7.6 years (median 17 years; range 5‐26 years) and the mean age at diagnosis was 23 ± 8.4 years (median 24 years; range 14‐40 years). From the initial presentation to the confirmation of diagnosis, there was a significant delay of 11.7 years. Renal impairment was the predominant manifestation in 38/54 (70.3%). The commonest symptom of FD in this cohort was neuropathic pain in 34/54 (62.9%).

**TABLE 1:** Demographic and clinical parameters of 54 Fabry patients

Total number of patients	n (54)
*Sex*	
Male	44
Female	10
*Consanguinity*	4 families
Number of patients on ERT	28
Agalsidase beta	21
Agalsidase alfa	7
Average age of starting ERT (n‐28) (mean, range in years)	18.63 ± 15.73 y (range 0‐58 y)
*Age at first symptom*	
5‐10 y	9
10‐20 y	21
20‐30 y	7
30‐40 y	3
40‐50y	3
51‐60 y	1
Age not available	3
Asymptomatic	7
*Age at diagnosis*	
<10 y	1
10‐20 y	16
20‐30 y	11
30‐40 y	11
40‐50 y	8
50‐60 y	7
*Symptom at presentation*	
Renal failure	19
Angiokeratoma	3
Neuropathic pain	12
Stroke	5
Hypertrophic cardiomyopathy	4
Mutation analysis	49/54 (90.7%)
Average delay in diagnosis (from age of first symptom to age of diagnosis) (mean in y)	11.7 y
*Duration of follow‐up*	
<1 y	14
1‐5 y	21
5‐10 y	9
10 y or >10	4
Lost for follow‐up	3
Expired	3
*Neuropathic pain*	
Yes	34/54 (62.9%)
No	17
NA	3
Average age of onset (n‐29) (mean, range in years, median)	13.67 ± 10.17 y (range 5‐32 y; median 9 y)
Number of patients using pain medications (carbamazepine (n = 6), gabapentin (n = 4), paracetamol (n = 3), NSAID (n = 1), liofen (n = 1) and pregabalin (n = 1))	16/32 (50%)
*Angiokeratoma*	
Yes	27/52 (51.9%)
No	25
NA	2
Average age of angiokeratoma (n‐20),(mean, range in years, median)	16.67 ± 7.17 y (range 6‐25 y; median 17 y)
*Hypohydrosis*	
Yes	21/50 (42%)
No	29
NA	4
Interference with activity	15/19
Number of patients with hypohidrosis on ERT	15
Improvement with ERT (n‐15)	10/15
*Cornea verticillata*	
Yes	27/39 (69.2%)
No	12
NA	15
*Kidney dysfunction*	
Yes	38/54 (70.3%)
No	16
Average age of onset (n‐37), (mean in years, range, median)	23.67 ± 7.649 y (range 17‐40 y; median 20 y)
*CGA staging (eGFR calculated with MDRD)*	
G1	20
G2	15
G3a	2
G3b	2
G4	3
G5	8
NA	4
Gross proteinuria	18/54 (33%)
Renal biopsy	18
Dialysis	5
Renal transplantation	4
Microalbuminuria (30‐300 mg/day)	20/54 (37%)
ACE inhibitors	16/37
Average age of dialysis	31.4 y
*Cardiac symptoms*	
Echocardiogram done	49/54 (90.7%)
Normal	26/49 (53%)
Left ventricular hypertrophy	22/49 (44.8%)
Average age of onset (n‐16)	36.4 y
Arrhythmia	4/54 (7.4%)
Complete heart block	3/54 (5.5%)
Pacemaker implantation	3/54
Atrial fibrillation	1/54
Echo not done	5
Hypertension	13
*Stroke*	5
Recurrent stroke	4/5
Stroke as presenting symptom	5
Ischemic	3
NA	2
*Abdominal symptoms*	23/54
Pain	4
Diarrhea/urgency	10
Constipation	5
Nausea	2
Vomiting	2
Patients on ERT with abdominal symptoms	11
Improvement after ERT	6/11 (54.5%)
Weight gain with ERT	11/28
*Hearing loss*	
Yes	13
No	25
NA	16
*Females with Fabry*	
Total (n)	10
Symptomatic	2/10
End stage renal failure	1
Stroke	1
Asymptomatic	8/10
Cornea verticillata	8/10 (not tested‐1)
Microalbuminuria	4/10
Echo	
Normal	8/10
LVH	2/10
Neuropathic pain	5/10
Hypohydrosis	None
Expired	4/54 (three males, one female)

Abbreviations: LVH, Left ventricular hypertrophy; MDRD, Modification of Diet in Renal Disease Study; NSAID, nonsteroidal anti‐inflammatory drug.

Of the 49/54 patients who had ECG and echocardiogram data, 44.8% of patients (22/49) had evidence of left ventricular hypertrophy (LVH) (Supplementary Figure S1D) and 26/49 (53%) had normal echocardiogram. The mean left ventricular ejection fraction (LVEF) was 59.4 ± 6.5% (36%‐70%). Three patients aged 35, 49 and 53 years were implanted with a dual chamber pacemaker for complete heart block. Another patient aged 41 years had atrial fibrillation and mild left ventricular outflow tract obstruction with normal LVEF.

Only 5/54 (9.2%) presented with stroke. Of these, four had an ischemic stroke. Angiokeratomas were present at the time of diagnosis in 27/52 (51.9%) (Supplementary Figure S1A) and hypohidrosis for 21/50 (42%). Slit lamp examination revealed cornea verticillata in 27/39 (69.2%) (Supplementary Figure S1B). The most common gastrointestinal symptom, postprandial diarrhea and urgency were present in10/23 (43.4%) patients. Ten patients (10/54; 18.5%) were female heterozygotes, of whom two were symptomatic. One female had biopsy and mutation‐proven FD (c.155G > A; p.C52Y), with end stage renal failure. The second symptomatic female was a 32‐year‐old woman with recurrent strokes and is currently receiving enzyme replacement therapy (ERT). X inactivation studies using HUMARA assay revealed skewed X inactivation. Her asymptomatic mother showed no skewing of the same X chromosome (Supplementary Figure S2). Nineteen patients (35%) were identified by family screening from twelve index cases. Then, 9/19 were symptomatic and ERT was initiated in 3 patients and the remaining received symptomatic treatment.

In addition, 33 *GLA* variants were identified in 49 patients, distributed across the 7 exons of *GLA* (18 missense variants, 5 nonsense variants and 10 frame shift mutations (8 deletions and 2 duplications). Supplementary Figure S1E illustrates the distribution of all the known and novel variants of the *GLA* gene. All 33 mutations were verified in The Human Gene Mutation Database (HGMD); of which 11 were novel mutations. For all the known and novel missense mutations, further analysis was done to estimate the effect of the mutations (supplementary material).

Since 2007, ERT was initiated in 28/54 (51.8%) patients. Four patients (4/54; 7.4%) on ERT have died. Three patients died from progressive renal failure (two males aged 31 and 44 years and a 25‐year‐old female). The third patient was a male who presented with recurrent stroke at 46 years of age.

## 4 DISCUSSION

We have presented data from 54 patients with confirmed FD, which is the largest cohort in India that we are aware of. We identified 33 *GLA* gene mutations in 49 patients with FD. In addition to 22 known mutations, we identified 6 novel missense mutations, 1 novel frameshift mutation, 3 novel deletions and 1 novel duplication in the *GLA* gene. Mutations were distributed in all the seven exons of the *GLA* gene but with no clear mutation hotspots. Most of the patients/families showed private mutations and we did not detect any recurrent mutations in *GLA* gene in Indian patients. We found 18 missense, 5 nonsense and 10 frameshift variants in our cohort. The mean age of onset of symptoms for the patients with missense mutations was 28.45 years whereas for the nonsense mutation group it was 12.5 years, and for frameshift variants, the age of onset was 11.2 years, demonstrating that loss of function variants (nonsense and frameshift) had earlier age of onset. Furthermore, all patients with loss of function variants (15 variants) showed <5% or undetectable levels of α‐gal A activity and presented as classic phenotype whereas only 10 out of 18 missense variants showed the nonclassic phenotype (Table 2). Thus, we could establish genotype‐phenotype correlation with respect to variant effects and severity of phenotype and this information could be helpful in genetic counseling for families with FD. In our cohort, the type of mutation alone was insufficient to stratify classical vs nonclassical variants, as there was significant variations observed in patients with the same mutation and between patients within the same family, probably due to disease modifiers or skewed X inactivation.

**TABLE 2:** Mutation spectrum and ACMG prediction, age of presentation and genotype phenotype correlation of our cohort

Type	Type of mutation/case	Number of patients	Age of first symptom	Age at diagnosis	Phenotype	Sex	Presentation	ACMG prediction	Enzyme level	Reference range	Protein domain	Position
Missense mutations	*GLA:c.59C > A:p.Ala20Asp*	1	28	34	C	M	Renal	Likely pathogenic	0.4nmol/hr/mg	0.8	NA	Ex1
	*GLA*:c.155G > A:p.Cys52Tyr	1	23	25	C	F	Symptomatic female with renal failure, expired	Likely pathogenic	1.298 pmol/punch/h	16.5‐59 pmol/punch/h	Dom_1	Ex1
	*GLA*:c.283T > C:pTrp95Arg	1	NA	28	C	M	Angiokeratoma	Likely pathogenic	2.3 nmol/h/ng	55‐85	Dom_1	Ex1
	*GLA*:c.335G > A:p.Arg112His	1	47	48	NC	M	Renal	Likely pathogenic	10.20 pmol/L/h	15.5‐58.5 pmol/punch/h	Dom_1	Ex2
	*GLA*:c.409G > T; p.Val137Phe	4	13	15	C	M	Angiokeratoma	Likely pathogenic	8.77 nmol/h/mg	45‐85	Dom_1	Ex3
			15	37	C	M	Stroke		8.3 nmol/h/mg	45‐85		
			15	35	C	M	Family screening		8.52 nmol/h/mg	45‐85		
			Asymptomatic	39	C	F	Family screening		Not available	NA		
	*GLA*:cc.413G > A:p.Gly138Glu	2	20	25	C	M	Renal	Likely pathogenic	0.1 μmol/L/h	(>2.6)	Dom_1	Ex3
			Asymptomatic	58	C	F	Family screening		Female carriers, did not do	NA		
	*GLA*:c.494A > G:p.Asp165Gly	1	24	24	C	M	Renal	Likely pathogenic	4 nmol/h/mg	45‐85	Dom_1	Ex3
	*GLA*:c.c.548G > T:p.G183V	2	6	22	C	M	Neuropathic pain	Likely pathogenic	2.4 nmol/h/mg	48 nmol/h/mg	Dom_1	Ex3
			6	14	C	M	Neuropathic pain		2.6 nmol/h/mL	48 nmol/h/mg		
	*GLA*:c.627G > T:p.Trp209Cys	1	39	40	C	M	Renal	Likely pathogenic	0.09 nmol/h/mg	22‐85 nmol/h/mg	Dom_1	Ex4
	*GLA*:c.657C > G p.Ile219Met	1	52	52	NC	M	Renal	Likely pathogenic	5.25	45‐85	Dom_1	Ex5
	*GLA*:c.668G > A:p.Cys223Tyr	1	17	40	C	M	Renal	Likely pathogenic	0.55	3‐20	Dom_1	Ex5
	*GLA*:c.680G > A:p.Arg 227Gln	1	29	30	C	M	Renal	Likely pathogenic	0.1 μmol/L blood/H	1.37‐7.66 μmol/L blood/hr	Dom_1	Ex5
	*GLA*:c.797A > G:p.Asp266Gly	1	32	33	C	M	Renal	Likely pathogenic	<0.062 nmol/h/mg	22‐85 nmol/h/mg	Dom_1	Ex5
	*GLA*:c.851T > C:p.Met284Thr	3	47	47	NC	M	Hypertrophic cardiomyopathy, Renal	Likely pathogenic	0.5 nmol/h/mg	3‐20 nmol/h/mg	Dom_1	Ex6
			Asymptomatic	54	NC	F	Family screening		1.9 nmol/h/mg	3‐20 nmol/h/mg		
			Asymptomatic	14	NC	M	Family screening		0.65 nmol/h/mg	3‐20 nmol/h/mg		
	*GLA*: c.902G > A:p.Arg301Gln	3	39	49	NC	M	Hypertrophic cardiomyopathy	Likely pathogenic	0.74 nmol/h/mL	3‐20 nmol/h/mg	Dom_1	Ex6
			52	43	NC	M	Hypertrophic cardiomyopathy		0.70 nmol/h/mL	3‐20 nmol/h/mg		
			Asymptomatic	18	NC	F	Family screening		Not done	Not done		
	*GLA*:c.1025G > A, p.Arg342Gln	1	13	19	C	M	Hearing loss, paraesthesia, mild protinuria	Likely pathogenic	0.1 nmol/h/mg	13‐67	Dom_2	Ex7
	*GLA*:c.1088G > A:p.Arg363His	1	NA	59	NC	M	Renal	Likely pathogenic	0.43	22‐85	Dom_2	Ex7
	*GLA*:c.640‐801G > A	1	52	52	NC	M	Hypertrophic cardiomyopathy	Likely pathogenic	4.5 μmol/L/h	≥15.3 μmol/L/h	NA	Int
Nonsense mutations	*GLA*:c.612G > A:p.Trp204term	1	25	28	C	M	Renal	Likely pathogenic	0.0 nmol/h/mg	22‐98.4 nmol/h/mg	Dom_1	Ex4
	*GLA*: c.658C > T:p.Arg220Term	1	11	25	C	M	Stroke	Likely pathogenic	<0.062 nmol/h/mg	22‐85 nmol/h/mg	Dom_1	Ex5
	*GLA*:c.679C > T:p.Arg 227Term	2	11	13	C	M	Neuropathic pain	Likely pathogenic	0.13 nmol/h/mg	13‐67	Dom_1	Ex5
			Asymptomatic	33	C	F	Family screening		Female carriers, did not do	NA		
	*GLA*:c.707G > A:p.Trp236Term	2	7	13	C	M	Neuropathic pain	Likely pathogenic	0.1 μmol/L/h	2.0‐14.6 μmol/L/h	Dom_1	Ex5
			10	12	C	M	Family screening		0.02 nmol/h/mg/L/h	13‐67 nmol/h/mg		
	*GLA*:c.1156 C > T:p.Gln386Term	1	11	14	C	M	Neuropathic pain	Likely pathogenic	0.4 nmol/h/mL	3‐20 nmol/h/mL	Dom_2	Ex7
Small deletions	*GLA*:c.25delC	2	15	35	C	M	Stroke	Likely pathogenic	0.1 nmol/h/mg	13‐67	Dom_1	5′ UTR
			10	8	C	F	Family screening		NA	NA		
	*GLA*:c.361_364delGCTA	2	6	19	C	M	Renal	Likely pathogenic	6.55 pmol/punch/h	usual affected range 6‐12.3	Dom_1	Ex2
			8	31	C	M	Renal		0.5 nmol/h/mg	13‐67		
	*GLA*:c.451_453delTAC	1	9	14	C	M	Neuropathic pain	Likely pathogenic	0.25 nmol/h/mL	3‐20 nmol/h/mL	NA	Ex3
	*GLA*:c.782delG:p.Gly261Valfs*8	1	10	36	C	M	Pain crisis, protinuria	Likely pathogenic	0.06 μmol/h/mg	>1.2 μmol/h/mg	Dom_1	Ex5
	*GLA*:c.1176delG	1	21	26	C	M	Renal	Likely pathogenic	0.8	3‐20	Dom_2	Ex1
	*GLA*:c. 1235_1236 del: p.Thr412serfs	3	18	27	C	M	Renal	Likely pathogenic	0.2	1.8‐7.6	Dom_2	Ex7
			8	32	C	F	Symptomatic female with stroke		2.6 nmol/h/mg	13‐67		
			10	55	C	F	Family screening		3.48 nmol/h/mg	3‐20 nmol/h/m		
	*GLA*:g.9356_9357delCA	1	19	25	C	M	Renal	Likely pathogenic	0.1 nmol/h/mg	NA	Dom_2	IVS6 ‐2
	*GLA*:c.1285_1285 del C:p.Leu429Phefs	1	10	41	C	M	Renal	Likely pathogenic	0.36 nmol /h/mL	3‐20 nmol/h/mL	Dom_2	Ex7
Duplications	*GLA*:c.270dupC	1	19	24	C	M	Renal	Likely pathogenic	0.35 nmol/h/mL	3‐20 nmol/h/mL	Dom_1	Ex2
	*GLA*:c.683dupA	2	5	16	C	M	Neuropathic pain	Likely pathogenic	0.23 nmol/h/mg	3‐20	Dom_1	Ex5
			Asymptomatic	41	C	F	Family screening		Female carriers, did not do	NA		
Unsolved	Lost to follow‐up	4				NA	NA		NA	NA	NA	NA
	Expired	1				NA	NA		NA	NA	NA	NA
	Total number of sample analyzed	54										

*Note*: Highlighted rows signify novel mutations. C, classical; NC, nonclassical; NA, not available; Dom_1 and Dom_2 as per supplementary Table S3.

Abbreviation: HCM, hypertrophic cardiomyopathy.

However, we also performed a detailed genotype‐phenotype correlation with the criteria used for classifying classical vs nonclassical phenotype in males and females.^5^ In our cohort of 54 patients, 40 patients had classical and 10 had nonclassical mode of presentation. Mutation results were unavailable for 5/54 but one among them was classified as classical as three of his siblings and a nephew had classical FD with proven mutation. There were four consanguineous families in our cohort. Only three families have undergone mutation analysis and one family was lost for follow‐up. They had the following mutations (*GLA*:c.409G > T;p.Val137Phe, *GLA*:c.494A > G:p.Asp165Gly, *GLA*:c. 1235_1236 del: p.Thr412serfs).

There was a significant diagnostic delay (11.7 years), and this is similar to previous published studies with diagnostic delays of up to 15 years.^2^ The reasons for delayed diagnosis include, significant heterogeneity in clinical presentations; poor genotype‐phenotype correlations; late‐onset presentations (cardiac and renal variants); variable presentations within extended family members (stroke, renal failure, cardiomyopathy); high prevalence of variants of unknown significance and polymorphisms.^6^, ^7^, ^8^ A three‐generation pedigree analysis is important to identify at‐risk relatives. Cascade screening provides an opportunity to identify pre‐symptomatic relatives. Counseling sessions should address psychosocial issues such as anxiety, guilt, grief, hopelessness and impact on self‐esteem.^9^

Renal impairment was found in 70.3% (38/54) of our FD patients. Gross proteinuria was evident in 18/54 (33%) whereas microalbuminuria was present in 37% in our cohort reiterating the need for close monitoring for evolution of renal impairment. The prevalence of FD was 0.87% among patients with chronic kidney disease (CKD) on dialysis.^10^ The prevalence of FD among those with CKD not on dialysis was only 0.2%.^6^ Renal impairment begins with microalbuminuria by the second or third decade of life in the classic phenotype. The patients should be regularly monitored with urine albumin/creatinine ratio and with eGFR. ACE inhibitors and angiotensin receptor blockers reduce proteinuria in patients with microscopic albuminuria. ERT is beneficial in the early stages of renal dysfunction. Transplanted kidneys remain free of Gb3 accumulation.

Neuropathic pain was the second commonest presentation. One should consider FD in patients presenting with neuropathic pain precipitated by fever, exercise, stress, changes in temperature and fatigue. In 10% to 20%, the pain stops with advancing age.^11^ Two of our patients, aged 12 and 15 years were suspected to have a functional disorder. A small fiber neuropathy could be confirmed on nerve conduction studies. Carbamazepine, gabapentin, phenytoin and pregabalin could be beneficial for neuropathic pain management. Nonsteroidal anti‐inflammatories should be avoided due to the risk of renal toxicity.

Cardiac involvement in FD is detected in more than 60% of affected males and heterozygote females. It includes myocarditis, hypertrophic cardiomyopathy (HCM), myocardial fibrosis, arrhythmias and cardiac failure.^12^ The prevalence of FD in patients with HCM is estimated to be 0.94% and 0.90% of male and female cardiac patients screened.^13^Concentric LVH, which is typically nonobstructive, is seen in 50% of males and one‐third of females.^13^ LVH in females is delayed by around 10 years than males but scarring has been observed even in the nonhypertrophied stages.^14^ Arrhythmias are reported in 27% to 42% males and 27% of females with FD, and may cause sudden cardiac deaths. Atrioventricular blocks may benefit from cardiac pacing or implantation of defibrillators. Out of the 49 patients who had echocardiogram data, 44.8% of patients (22/49) in our cohort had evidence of LVH on the echocardiogram. Only five patients (5/49) were found to have left ventricular systolic dysfunction (10.2%). One patient (1/49; 2.04%) was documented to have left ventricular outflow tract obstruction. Four (4/54; 7.4%) patients had arrhythmia and three of them were implanted with dual chamber pacemaker for complete heart block and one patient had atrial fibrillation. Cardiac magnetic resonance imaging accurately quantifies left ventricular mass. Late gadolinium enhancement in the basal posterolateral segments is classically observed in FD patients with myocardial fibrosis.^14^ Cardiac MRI is not routinely performed in India for Fabry patients but 3/54 patients underwent cardiac MRI in our cohort and late gadolinium enhancement was observed in all three of them. ERT is recommended when there is increased left ventricular wall thickening >12 mm.^15^ ERT facilitates reduction in LVH in areas without myocardial fibrosis.

Cerebrovascular involvement in FD includes headache, vertigo, transient ischemic attack (TIA) and ischemic stroke. FD patients have 5.5‐ to 12.2‐fold increased risk of stroke compared to the general population.^4^ A large analysis of 63 *GLA* screening studies showed prevalence of FD among 0.13% and 0.14% of male and female stroke patients screened.^13^ In our cohort, 5/54 (9.6%) had history of stroke. Hyperintensity of the pulvinar suggesting calcification (pulvinar sign) is specific for FD (Supplementary Figure S1C). Dolichoectasia of the vertebrobasilar circulation should prompt the clinicians to screen for underlying FD.^2^ Patients with stroke or TIA should be started on a combination of enteric‐coated aspirin, clopidogrel and statins.^16^

Angiokeratomas cluster in the bathing trunk region and around the umbilicus. They are seen in around 40% of male patients with classic disease by the age of 14 to 16 years.^17^ Angiokeratoma has been reported as early as 4 years of age.^18^ “Late onset” variants usually lack angiokeratoma, hypohidrosis and acroparesthesia, which lead to further delay in diagnosis.^19^ Abdominal pain is mainly precipitated postprandially and can manifest as early as 1 year of age.^18^ Hypohidrosis significantly interferes with the quality of life due to reduced exercise tolerance. Female patients with FD have hyperhidrosis.^18^ Angiokeratoma, gastrointestinal symptoms, hypohidrosis are often early clinical manifestations in the classic phenotype. In our cohort, these findings did not lead to an early diagnosis of FD, emphasizing the importance of disease awareness initiatives in India. Depression is an important underrecognized problem in FD. Adolescent patients usually have chronic fatigue. Para‐pelvic kidney cysts are seen in around 50% of patients and this clue helps in the early recognition of FD.^2^

The common misdiagnoses in male and female patients with FD include systemic lupus erythematosus, growing pains (frequent misdiagnosis in children), peripheral neuropathy, chronic fatigue syndrome and hypochondriasis.^20^ Coeliac disease and multiple sclerosis are also often cited as differential diagnoses in females.^2^

Then, 60% to 70% of female heterozygotes have some disease manifestation and around 10% have severe manifestations.^20^, ^21^ It is important to monitor females with FD during their lifetime. In our cohort, 50% of the female heterozygotes had disease manifestations. The number of affected females with FD for every proband was approximately twice the number of affected males.^20^

In our cohort of 54 patients with FD, only 10 patients had features consistent with “nonclassic” phenotype. The low prevalence of nonclassic variants in our cohort is probably due to the lack of high risk screening protocols for identifying Fabry patients in India. This study highlights the importance of enzyme analysis and slit lamp examination for cornea verticillata as cost‐effective tools for early detection of FD among patients with idiopathic CKD, HCM and cryptogenic stroke.

Family screening is the most efficient way to identify asymptomatic relatives.^21^ On an average five family members are diagnosed for every proband with FD.^20^ A 6 year study from Spain identified 77 new patients among which 51 (51/77; 66.2%) were identified through family screening.^1^ In our cohort only 19/54 (20.3%) of patients were identified following family screening.

The hurdles to successful screening in India could include (a) the fear of being diagnosed with a chronic disease; (b) social taboos in India related to chronic diseases; (c) the cost of disease modifying treatments such as ERT; and (d) the lack of access to ERT, with Government funded ERT currently available only in Karnataka, which is one of 28 Indian States.

Only 17% of individuals identified by screening have classical FD phenotype.^22^ The majority have a late onset presentation due to a variant of unknown significance (VUS) in *GLA*. Lyso Gb3 values of >1.3 nmol/L confirms the diagnosis of FD in individuals with a VUS in *GLA* with nonclassical phenotype.^22^ Newborn screening (program is useful to pick up FD.^23^ Prenatal diagnosis is possible, but the family should be counseled regarding the intrafamilial variability and late onset variants.

Commercially two ERTs are available in India; agalsidase alfa (Replagal, Takeda) (0.2 mg/kg biweekly) and agalsidase beta (Fabrazyme, Genzyme) (1 mg/kg biweekly).^24^ ERT was initiated for 28/54 (51.8%) in our cohort through charitable programs. The average age of initiation of ERT was 27.3 years. The proposed guidelines for initiation of ERT is soon after the confirmation of the diagnosis for males >16 years, by 8 to 10 years for boys with classic FD, and at the time of progression to organ involvement in females at any age.^14^, ^17^ Migalastat, a pharmacological chaperone, is approved in a few countries as an oral monotherapy and is an alternative to ERT in FD patients with specific amenable mutations^25^ but is not approved in India.

The limitation of this study included restrospective data collection and lack of standardized documentation of improvements in clinical parameters after ERT initiation. This Indian Fabry cohort provided us with an unique opportunity to understand the disease burden, its varied clinical presentation and significant delay in diagnosis. We identified several novel pathogenic mutations in our cohort. Our study has highlighted the importance of FD being included in the panel of investigations for idiopathic CKD, HCM and cryptogenic stroke. This offers disease‐modifying treatment opportunities for the proband and facilitates early detection of asymptomatic FD patients through family screening.

## CONFLICTS OF INTEREST

S. N. is an advisory member of INCAP. S. N. has received travel grants for INCAP advisory board meetings. S. N. has no conflict of interest related to this manuscript. D. Y. does not have any conflict of interest for the study/paper. A. B. does not have any conflict of interest for the study/paper. H. A. has no conflicts of interest related to this manuscript. R. D. P. access to ERT is provided under the Indian Charitable Access Program (INCAP) of Sanofi Genzyme for patients of authors R. D. P. The association is purely academic, and no financial compensation is provided. The author is also committee members of the INCAP /CAP, Takeda programme. The author declares no other conflicts of interest. N. G. has no conflicts of interest related to this manuscript. M. K. does not have any conflict of interest for the study/paper. P. R. has no competing interests or conflict of interest to declare with reference to the paper on FD submitted to JIMD Reports M. B. is an honorary member of Indian Medical Advisory Board, Sanofi‐Genzyme, India. M. B. has no conflict of interest related to this manuscript. S. P. does not have any conflict of interest, except that her patients had received and have been receiving ERT for FD under India Charitable Access Program of Sanofi‐Genzyme and Shire Charitable Access Program. R. R. D. does not have any conflict of interest for the study/paper. S. J. is an advisory member of INCAP. S. J. has no conflict of interest related to this manuscript. S. D. is a member of the Indian Medical Advisory Board Member (IMAB) and has no conflict of interest with regard to this manuscript. P. S. does not have any conflict of interest for the study/paper. K. M. does not have any conflict of interest for the study/paper. S. B. M. does not have any conflict of interest for the study/paper. R. M. is the Medical director and employee at Sanofi Genzyme India. I. C. V. does not have any conflict of interest for the study/paper. A. D. is a nonpaid member of the Scientific Advisory Committee of Sanofi Genzyme. A. D. has no conflict of interest related to this manuscript. U. R. is an International advisory member of INCAP and Medical Expert Committee member for Takeda's Compassionate Access Programme (CAP). U. R. has received travel grants for INCAP advisory board meetings. U. R. has no conflict of interest related to this manuscript.

## AUTHOR CONTRIBUTIONS

**Sheela Nampoothiri** conceived and with **Uma Ramaswami** prepared a core data set for all clinicians. **Sheela Nampoothiri** and **Dhanya Yesodharan** prepared the draft manuscript and performed detailed literature searches. **Hisham Ahamed**, **Ratna Dua Puri**, **Neerja Gupta**, **Madhulika Kabra**, **Prajnya Ranganath**, **Meenakshi Bhat**, **Shubha Phadke**, **Radha Rama Devi Akella**, **Sujatha Jagadeesh**, **Sumita Danda**, **Padmavathy Narayana Sylaja**, **Kausik Mandal**, **Sunita Bijarnia‐Mahay**, and **Ishwar Chander Verma** enrolled patients and provided detailed clinical and demographic information and critically analyzed the paper and provided suggestions. **Shubha Phadke** and **Kausik Mandal** performed molecular analysis of 14 patients with FD. **Ashwin Dalal** and **Amrita Bhattacherjee** prepared the molecular aspects of the manuscript. **Hisham Ahamed** conducted a detailed literature search and contributed to the cardiological aspects of the cohort. **Uma Ramaswami**, **Ishwar Chander Verma**, and **Ravinder Makkar** have critically evaluated the manuscript and had contributed suggestions.

## PATIENT CONSENT

Informed consent was obtained from all patients for being included in the study.

## ETHICS APPROVAL

As this is a retrospective analysis, ethics approval was not needed.

## Supporting information


**Appendix S1**. Supporting Information.

**Supplementary Figure S1(a)** Clustering of angiokeratomas over the abdomen **(b)** slit lamp evaluation showing cornea verticillata **(c)** hyperintensity due to calcification of the pulvinar region in T2W image; thalamus (thin arrow) and globus pallidus and putamen (thick arrow) **(d)** Apical four chamber view demonstrating concentric hypertrophy of the left and right ventricle and interventricular septum (thick arrow), lateral wall of left ventricle (LV) (thin arrow) **(e)** Mutations hotspots are highlighted and depicted in the *GLA* gene using various colour codes. Novel mutation (missense mutations, deletions and duplications) distributed in exonic and intronic regions are highlighted in orange colour, known variants are depicted in separate colours.

**Supplementary Figure S2** HUMARA assay for X inactivation study shows random X inactivation in mother as evidenced by decrease in peak size of 283 bp PCR product after HpaII digestion. However in patient there is disproportionate reduction of peak size for 283 bp product suggestive of skewed X inactivation.

**Supplementary Figure S3** Chain A of protein 1R46, showing two domains of the protein, Melibiase_2 (Blue) and Melibiase_2_C (Magenta), 16 missense mutations shown in the domain regions of the protein, Novel mutations are colour coded with yellow and known ones are in orange.

**Supplementary Figure S4**: Novel missense mutations c.283 T > C:p.W95R,c.409G > T:p.V137F, c.627G > T:p.W209C, c.657C > G p.I219M and c.797A > G:p.D266G are shown in protein structure in the figure using In silico studies showing changes in inter molecular interactions and bonding using various colour codes.

**Supplementary Table S1** Represents the population database reports and prediction scores for the known deletion, duplication and frameshift mutations identified in patients in *GLA* gene

**Supplementary Table S2** Mutation effect on the protein stability is predicted using protein pdb 1R46 protein structure

**Supplementary Table S3** Protein 1R46 Pfam domain details
