# Supplementary material for: Fabry disease in India: A multicenter study of the clinical and mutation spectrum in 54 patients
Source: JIMD Rep. 2020 Aug 15;56(1):82–94. doi: 10.1002/jmd2.12156 (PMC7653245; doi:10.1002/jmd2.12156)
Supplement: Supplementary file 1 — Appendix S1. Supporting Information. [file JMD2-56-82-s001.docx]

**Supplementary material**

**Methodology**

**Molecular characterization of GLA gene**

Genomic DNA from whole blood was extracted using the phenol chloroform extraction method. Primers were designed for all seven exons and the flanking exon- intron boundaries (100 bases) of the *GLA* gene using Primer 3 Input version 0.4.0 and NCBI primer BLASTsoftware. Polymerase chain reaction (PCR) was carried out in the Bio-Rad DNA Engine Peltier Thermal Cycler (Bio-Rad Laboratories, Inc., Hercules, CA) for all seven exons of GLA with specific primers, for 30 cycles. Bidirectional sequencing was carried out on all the purified PCR products by capillary electrophoresis on ABI3130 automated genetic analyzer (Life Technologies, ThermoFisher Scientific Corporation, Foster City, CA). The sequencing data were analyzed using the software EMBOSS^1^ and ChromasLite. The Human Genome Variation Society (HGVS) nomenclature guidelines were used for reporting the identified sequence variations^2^ .

**In Silico Characterization of Identified Sequence Variants**

The variants identified in the patient samples , which can be classified intomissense mutations, deletion and duplications (CNVs) respectively are given in Table 2. Identified variations were investigated in various population databases such as 1000 genomes^3^ , ExAC^4^ , gnomAD^5^ , HGMD^6^ (Human Gene Mutation Database) , dbSNP^7^ , Clinvar^8^ and our In-house databases to evaluate the already known or unknown mutations and to identify whether a mutation was a polymorphism or not using a minor allele frequency (MAF) of ≤ 0.01.Variant interpretation followed ACMG guidelines to predict the pathogenicity of the variant and confirmed using in silico prediction tools like SIFT^9^ (Sorting Intolerant from Tolerant) , Mutation Taster 2^10^ , M-CAP^11^ and CADD^12^ (Combined Annotation Dependent Depletion) . Deletion and duplication cannot be accessed using all the tools, but Mutation taster 2 can evaluate the effect of these.

**Characterization of Effects of Novel Mutations on Protein Sequence and Structure**

In silico examination was also done to study the effect of the identified missense variants on protein stability.Various standard mutation stability prediction tools such as SDM ^13^(Site Directed Mutator) , mCSM^14^ (mutation Cutoff Scanning Matrix) and DUET^15^ were used for analysis. The effect of the variants on protein structure was reviewed using the already available structure 1R46 (Chain A) in PDB (Protein Data Bank) as reference^16^ . Pfam database^17^ is used to detect the domains present in the protein.

**Results**

Study in population databases interpreted the absence of the six novel missense mutations in 1000 genomes, ExAC, GenomAD, dbSNP, Clinvar population databases and were not previously described in our In-house databases. *GLA*: c.59C>A:p.Ala20Asp was predicted as damaging in SIFT, but Mutation Taster-2 predicted it as polymorphism and CADD score was 16.03 which clearly designated the mutation to be a polymorphism. All the variants were analysed based on the above - mentioned databases and as per the CADD score (>20), they were classified as disease causing and damaging.

Novel variants were classified as per the American College of Medical Genetics and Genomics (ACMG) and the Association for Molecular Pathology guidelines^18^. The interpretation of various population databases and prediction tools is given in Supplementary Table 1, providing information about the mutation analysis of the novel and known copy number variation (CNV), deletion, duplication and frameshift mutation of the *GLA* gene. All novel mutations in this study were predicted as ‘disease-causing’ and ‘likely pathogenic’ based on various database score cut offs except one missense mutation that was predicted as a polymorphism.

The effect of the variant on protein stability was examined through the in-silico stability testing tools SDM, mCSM, and DUET. As per the in-silico prediction tools, all 5 novel missense mutations were destabilizing the protein structure by predicting changes in folding free energy (ΔΔG) which leads us to conclude the damaging effect of the mutation. The output of these software is tabulated in Supplementary Table 2. The most identical structure of the protein from *GLA* transcript ENST00000218516.3 (429 aa) is 1R46 human alpha-galactosidase present in RCSB-PDB database as a homodimer with each monomer containing a (β/α) 8 domain with the active site and an antiparallel β domain. As the structure of the protein 1R46 chain A starts from amino acid number 36, we are unable to locate the polymorphic novel variant in the protein structure. Protein 1R46 has two domains; Alpha galactosidase A and Alpha galactosidase A C-terminal beta sandwich present in it as retrieved from Pfam database (Supplementary Table 3). Both these domains are from Melibiase_2 family domains. Alpha-galactosidase (EC 3.2.1.22) (melibiase) catalyzes the hydrolysis of melibiose into galactose and glucose. In human, the deficiency of this enzyme is the cause of FD. Alpha-galactosidase is present in a variety of organisms. Most of the mutations including the novel ones were present mostly in Pfam domain Alpha galactosidase A. As a point mutation in α-GAL can lead to FD, we plotted the location of 11 missense mutations in the protein structure. The Supplementary Figure 3 depicts domain distribution of the mutations in the protein structure. Novel missense mutations were further analyzed for intermolecular bonds within the protein structure. In-silco studies of the amino acid change for the novel mutations unequivocally demonstrated the change in the intermolecular ionic interactions and the change of inter-molecular hydrogen and ionic bond interactions of the protein structure, indicating protein structure destabilization; details represented in Supplementary Figure 4.

**References**

# 1. [Rice P](https://www.ncbi.nlm.nih.gov/pubmed/?term=Rice%20P%5BAuthor%5D&cauthor=true&cauthor_uid=10827456), Longden I, [Bleasby A](https://www.ncbi.nlm.nih.gov/pubmed/?term=Bleasby%20A%5BAuthor%5D&cauthor=true&cauthor_uid=10827456). EMBOSS: the European Molecular Biology Open Software Suite.[Trends Genet.](https://www.ncbi.nlm.nih.gov/pubmed/10827456) 2000;16:276-77.

2.Dunnen, Johan T. den, Stylianos EA. Mutation nomenclature extensions and suggestions to describe complex mutations: a discussion. Hum Mutat. 2000;15:7-12.

3.The 1000 Genomes Project Consortium.A global reference for human genetic variation.1000 Genomes Project Consortium.Nature.2015;526:68.

4. Karczewski KJ, Weisburd B, Thomas B, et al. The ExAC browser: displaying reference data information from over 60 000 exomes. Nucleic Acids Res. 2017;45: 840-45.

5. Karczewski, K. J., and L. Francioli. The Genome Aggregation Database (gnomAD). MacArthur Lab 2017.

6. Stenson PD, Ball EV, Mort M, et al. Human Gene Mutation Database (HGMD): 2003 update. Hum Mutat 21: 577–81.

7. [Sherry](javascript:;) ST, [Ward](javascript:;) MH, [Kholodov](javascript:;) M,  et al. dbSNP: the NCBI database of genetic variation. Nucleic Acids Res.2001;29: 308-11.

8.Landrum, Melissa J, Jennifer ML, et al. "ClinVar: public archive of interpretations of clinically relevant variants." Nucleic Acids Res.2015;44: 862-68.

9. Sim NL, Kumar P, Hu J, et al. SIFT web server: predicting effects of amino acid substitutions on proteins. Nucleic Acids Res. 2012 ;40: 452- 57.

10. Schwarz JM, Cooper DN, Schuelke M, et al. MutationTaster 2: mutation prediction for the deep-sequencing age. Nat Methods. 2014 ;11:361-62 .

11.Jagadeesh K, Wenger M, Berger M etal.M-CAP eliminates a majority of variants of uncertain significance in clinical exomes at high sensitivity. Nat Genet. 2016;48:1581–86.

12. Rentzsch, P, Daniela W, Gregory MC, et al. CADD: predicting the deleteriousness of variants throughout the human genome. Nucleic Acids Res .2018;47:886-94.

13.Catherine LW, Robert P, Tom LB. SDM- a server for predicting effects of mutations on protein stability and malfunction. Nucleic Acids Res.2011;39: 215-22.

14. Douglas EVP, David BA, Tom LB. mCSM: predicting the effects of mutations in proteins using graph-based signatures. Bioinformatics.2013;30:335-42.

15. Douglas EVP, David BA,Tom LB.DUET: a server for predicting effects of mutations on protein stability using an integrated computational approach. Nucleic Acids Res.2014;42:314-19.

16. Garman SC, David NG. The molecular defect leading to Fabry disease: structure of human a-galactosidase.[J Mol Biol.](https://www.ncbi.nlm.nih.gov/pubmed/15003450)2004;337: 319-35.

17. Alex B, Lachlan C, Richard D, et al. The Pfam protein families database. Nucleic Acids Res.2004; 32: 138-41.

18. Richards S, Nazneen A, Sherri B, et al. Standards and guidelines for the interpretation of sequence variants: a joint consensus recommendation of the American College of Medical Genetics and Genomics and the Association for Molecular Pathology. Genet Med.2015;17**:** 405.
