# Supplementary figures and images for: Fabry disease in India: A multicenter study of the clinical and mutation spectrum in 54 patients
Source: JIMD Rep. 2020 Aug 15;56(1):82–94. doi: 10.1002/jmd2.12156 (PMC7653245; doi:10.1002/jmd2.12156)

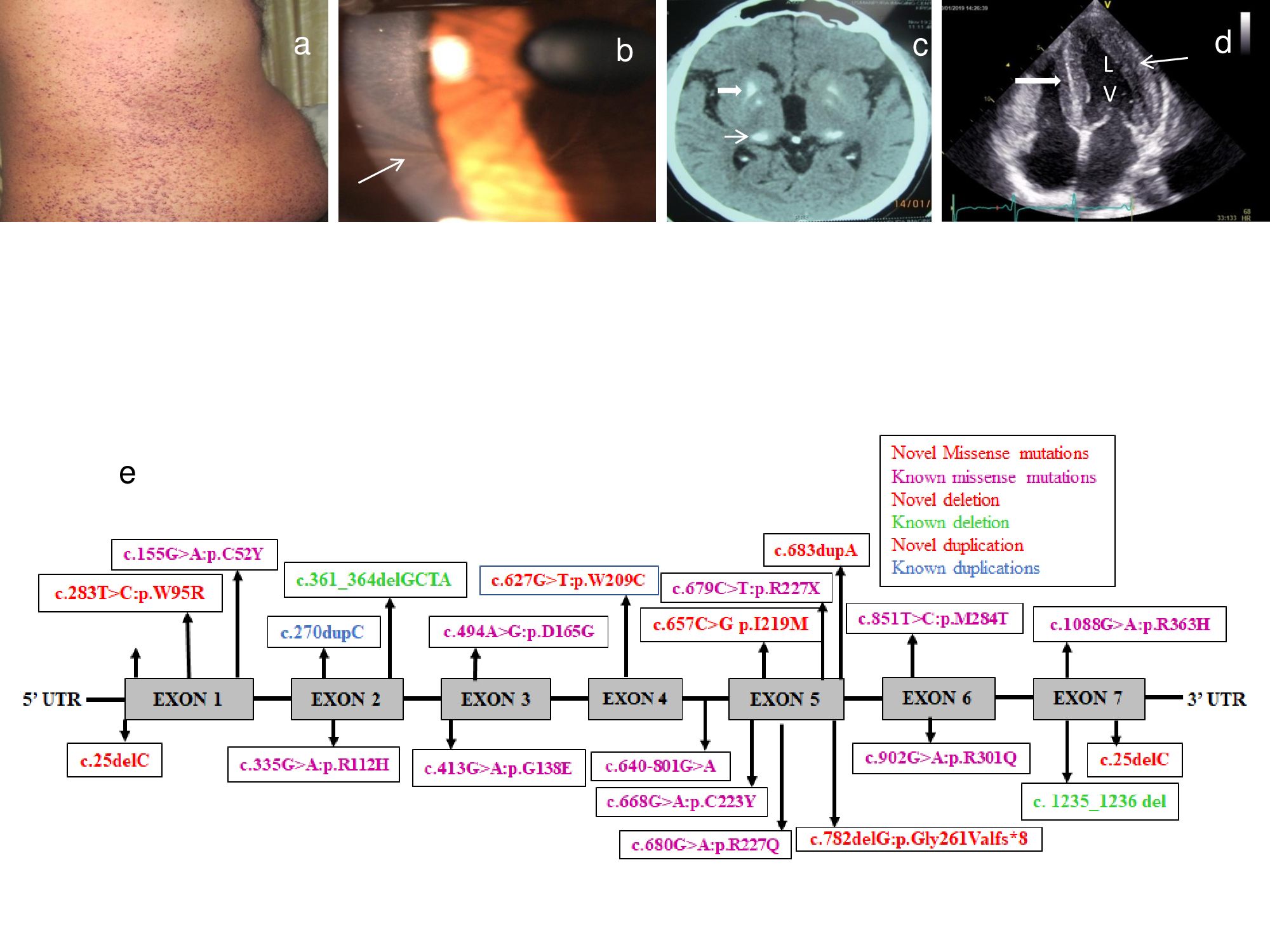

Supplement: Supplementary file 2 — Supplementary Figure S1(a) Clustering of angiokeratomas over the abdomen (b) slit lamp evaluation showing cornea verticillata (c) hyperintensity due to calcification of the pulvinar region in T2W image; thalamus (thin arrow) and globus pallidus and putamen (thick arrow) (d) Apical four chamber view demonstrating concentric hypertrophy of the left and right ventricle and interventricular septum (thick arrow), lateral wall of left ventricle (LV) (thin arrow) (e) Mutations hotspots are highlighted and depicted in the GLA gene using various colour codes. Novel mutation (missense mutations, deletions and duplications) distributed in exonic and intronic regions are highlighted in orange colour, known variants are depicted in separate colours. [file JMD2-56-82-s002.jpg]

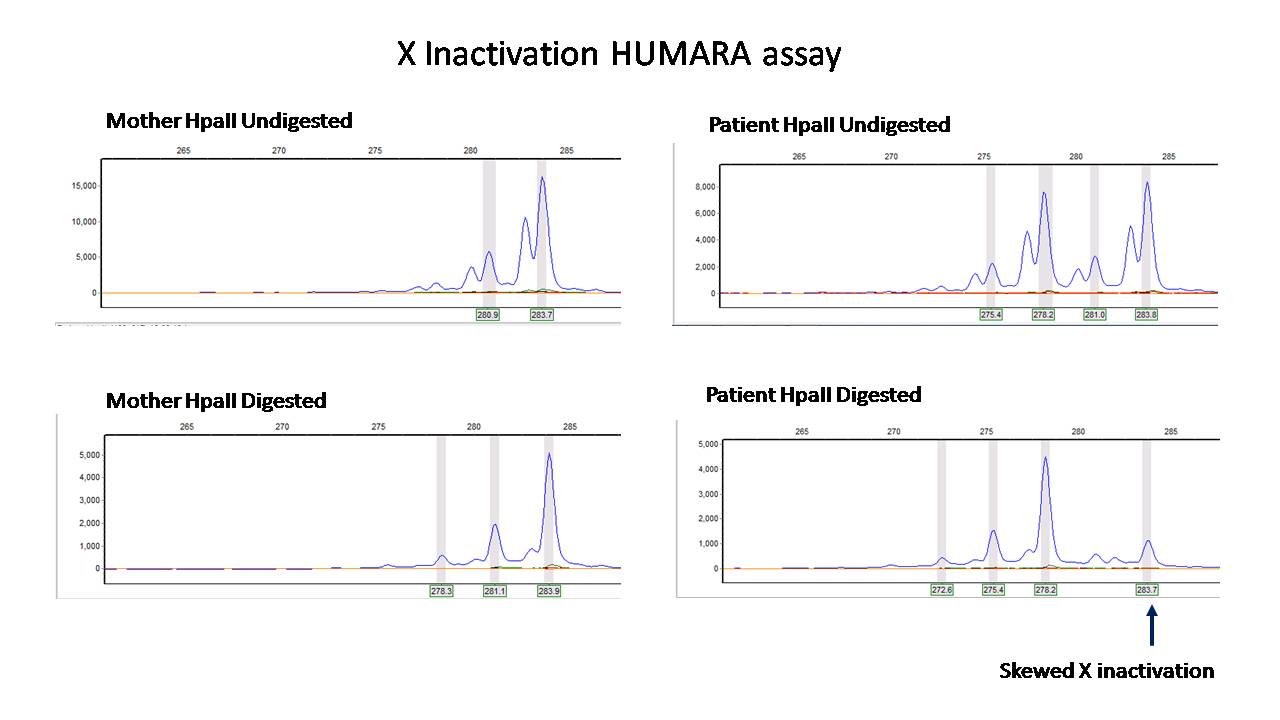

Supplement: Supplementary file 3 — Supplementary Figure S2 HUMARA assay for X inactivation study shows random X inactivation in mother as evidenced by decrease in peak size of 283 bp PCR product after HpaII digestion. However in patient there is disproportionate reduction of peak size for 283 bp product suggestive of skewed X inactivation. [file JMD2-56-82-s003.jpg]

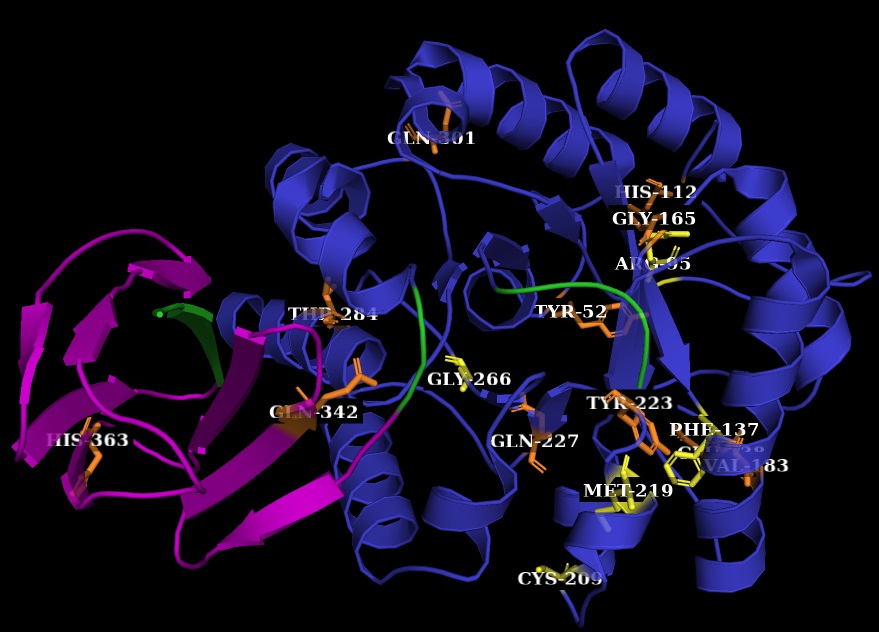

Supplement: Supplementary file 4 — Supplementary Figure S3 Chain A of protein 1R46, showing two domains of the protein, Melibiase_2 (Blue) and Melibiase_2_C (Magenta), 16 missense mutations shown in the domain regions of the protein, Novel mutations are colour coded with yellow and known ones are in orange. [file JMD2-56-82-s004.jpg]

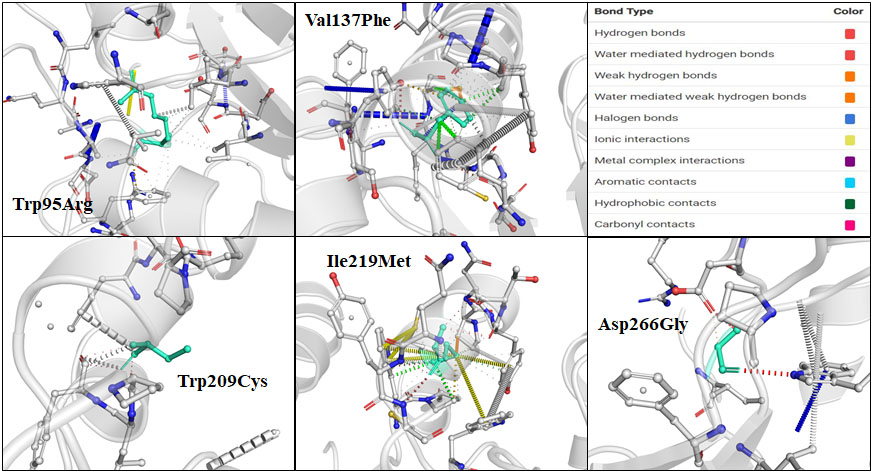

Supplement: Supplementary file 5 — Supplementary Figure S4: Novel missense mutations c.283 T > C:p.W95R,c.409G > T:p.V137F, c.627G > T:p.W209C, c.657C > G p.I219M and c.797A > G:p.D266G are shown in protein structure in the figure using In silico studies showing changes in inter molecular interactions and bonding using various colour codes. [file JMD2-56-82-s005.jpg]
