# Supplementary material for: Fabry disease in India: A multicenter study of the clinical and mutation spectrum in 54 patients
Source: JIMD Rep. 2020 Aug 15;56(1):82–94. doi: 10.1002/jmd2.12156 (PMC7653245; doi:10.1002/jmd2.12156)
Supplement: Supplementary file 6 — Supplementary Table S1 Represents the population database reports and prediction scores for the known deletion, duplication and frameshift mutations identified in patients in GLA gene [file JMD2-56-82-s006.docx]

**Supplementary Table 1**: Represents the population database reports and prediction scores for the known deletion, duplication and frameshift mutations identified in patients in *GLA* gene

| **Type** | **Variants** | **Database reports** | | | | | | | Predictions (scores) | | | | | |
| --- | --- | --- | --- | --- | --- | --- | --- | --- | --- | --- | --- | --- | --- | --- |
|  |  | **1000 genomes** | **ExAC** | **gnomAD** | **dbSNP** | **In-house database** | **Indian database** | **HGMD** | **ClinVar** | **SIFT** | **Polyphen-2** | **MutationTaster2** | **M-CAP** | **CADD** |
| **Missense Mutations** | ***GLA*:c.59C>A:p.Ala20Asp** | NA | NA | NA | Novel | NA | NA | NA | NA | Damaging | PD | disease causing | PP | 25.9 |
|  | ***GLA*:c.155G>A:p.Cys52Tyr** | NA | NA | NA | Novel | NA | NA | Reported | NA | Damaging | PD | disease causing | PP | 33 |
|  | ***GLA*:c.283T>C:pTrp95Arg** | NA | NA | NA | Novel | NA | NA | NA | NA | Damaging | PD | disease causing | PP | 28.9 |
|  | ***GLA*:c.335G>A:p.Arg112His** | NA | 1.14E-05 | NA | Novel | NA | NA | Reported | Pathogenic/Likely pathogenic | Damaging | PD | disease causing | PP | 32 |
|  | ***GLA*:c.409G>T; p.Val137Phe** | NA | NA | NA | Novel | NA | NA | NA | NA | Damaging | PD | disease causing | PP | 25.4 |
|  | ***GLA*:c.413G>A:p.Gly138Glu** | NA | NA | NA | Novel | NA | NA | Reported | NA | Damaging | PD | disease causing | NA | 28.9 |
|  | ***GLA*:c.494A>G:p.Asp165Gly** | NA | NA | NA | Novel | NA | NA | Reported | NA | Damaging | PD | disease causing | PP | 29.2 |
|  | ***GLA*:c.548G>T:p.Gly183Val** | NA | NA | NA | rs398123212 | NA | NA | Reported | Pathogenic​ | Damaging | PD | disease causing | PP | 24 |
|  | ***GLA*:c.627G>T:p.Trp209Cys** | NA | NA | NA | Novel | NA | NA | NA | NA | TOLERATED | PD | disease causing | PP | 22.6 |
|  | ***GLA*:c.657C>G:p.Ile219Met** | NA | NA | NA | Novel | NA | NA | NA | Likely pathogenic | TOLERATED | PD | disease causing | PP | 22.5 |
|  | ***GLA*:c.668G>A:p.Cys223Tyr** | NA | NA | NA | NA | NA | NA | Reported | NA | Damaging | PD | disease causing | PP | 29.1 |
|  | ***GLA*:c.680G>A:p.Arg 227Gln** | NA | NA | NA | rs104894840 | NA | NA | Reported | Pathogenic | Damaging | PD | disease causing | PP | 29.8 |
|  | ***GLA*:c.797A>G:p.Asp266Gly** | NA | NA | NA | Novel | NA | NA | NA | NA | Damaging | PD | disease causing | PP | 31 |
|  | ***GLA*:c.851T>C:p.Met284Thr** | NA | NA | NA | Novel | NA | NA | Reported | NA | Damaging | PD | disease causing | PP | 26.5 |
|  | ***GLA*: c.902G>A:p.Arg301Gln** | NA | NA | NA | rs104894828 | NA | NA | Reported | Pathogenic | Damaging | PD | disease causing | PP | 28.4 |
|  | ***GLA*:c.1025G>A, p.Arg342Gln** | NA | NA | NA | rs28935493 | NA | NA | Reported | Pathogenic​ | Damaging | PD | disease causing | PP | 29.9 |
|  | ***GLA*:c.1088G>A:p.Arg363His** | NA | NA | NA | rs111422676 | 0.996479 | 0.0006477 | Reported | NA | TOLERATED | BENIGN | polymorphism | PP | 9.622 |
|  | ***GLA*:c.640-801G>A** | NA | NA | 0.0000453 | rs199473684 | NA | NA | Reported | Uncertain significance | NA | NA | Disease causing | NA | NA |
| **Nonsense mutations** | ***GLA*:c.612G>A:p.Trp204term** | NA | NA | NA | Novel | NA | NA | Reported | NA | Damaging due to stop | NA | disease causing | NA | 38 |
|  | ***GLA*: c.658C>T:p.Arg220Term** | NA | NA | NA | rs727503949 | NA | NA | Reported | Pathogenic​ | Damaging | NA | disease causing | NA | 37 |
|  | ***GLA*:c.679C>T:p.Arg 227Term** | NA | NA | NA | rs104894841 | NA | NA | Reported | Pathogenic​ | Damaging due to stop | NA | disease causing | NA | 35 |
|  | ***GLA*:c.707G>A:p.Trp236Term** | NA | NA | NA | rs879254022 | NA | NA | Reported | Pathogenic | Damaging | NA | disease causing | PP | 38 |
|  | ***GLA*:c.1156C>T:p.Gln386Term** | NA | NA | NA | Not present | NA | NA | Reported | NA | Damaging | NA | disease causing | NA | 35 |
| **Small Deletions** | ***GLA*:c.25delC** | NA | NA | NA | NA | NA | NA | Not reported | NA | NA | NA | disease causing | NA | NA |
|  | ***GLA*:c.361_364delGCTA** | NA | NA | NA | NA | NA | NA | Reported | NA | NA | NA | disease causing | NA | NA |
|  | ***GLA:*c.451_453delTAC** | NA | NA | NA | NA | NA | NA | Not reported | NA | NA | NA | disease causing | NA | NA |
|  | ***GLA*:c.782delG:p.Gly261Valfs*8** | NA | NA | NA | NA | NA | NA | Not reported | NA | NA | NA | disease causing | NA | NA |
|  | ***GLA*:c.1176delG** | NA | NA | NA | NA | NA | NA | Not reported | NA | NA | NA | disease causing | NA | NA |
|  | ***GLA*:c. 1235_1236 del:p.Thr412serfs** | NA | NA | NA | NA | NA | NA | Reported | NA | NA | NA | disease causing | NA | NA |
|  | ***GLA:*g.9356_9357delCA** | NA | NA | NA | NA | NA | NA | Reported | NA | NA | NA | NA | NA | NA |
| **Duplications** | ***GLA*:c.270dupC** | NA | NA | NA | NA | NA | NA | Reported | NA | NA | NA | disease causing | NA | NA |
|  | ***GLA*:c.683dupA** | NA | NA | NA | NA | NA | NA | Not reported | NA | NA | NA | disease causing | NA | NA |

Note: Highlighted rows signifies novel mutations, NA – Not available (In this case not variant is not reported in that particular database); PD – Probably damaging; PP – Possibly pathogenic;
