# Supplementary material for: Fabry disease in India: A multicenter study of the clinical and mutation spectrum in 54 patients
Source: JIMD Rep. 2020 Aug 15;56(1):82–94. doi: 10.1002/jmd2.12156 (PMC7653245; doi:10.1002/jmd2.12156)
Supplement: Supplementary file 7 — Supplementary Table S2 Mutation effect on the protein stability is predicted using protein pdb 1R46 protein structure [file JMD2-56-82-s007.docx]

**Supplementary Table 2**: Mutation effect on the protein stability is predicted using protein pdb 1R46 protein structure

|  | **Predicted Stability Change (ΔΔG) (Kcal/mol)** | | | | | |
| --- | --- | --- | --- | --- | --- | --- |
| **Mutation** | **mCSM** | **Prediction** | **SDM** | **Prediction** | **DUET** | **Prediction** |
| *GLA*:c.155G>A:p.C52Y | -1.292 | Destabilizing | 0.22 | Stabilizing | -1.05 | Destabilizing |
| *GLA*:c.283T>C:p.W95R | -2.57 | Destabilizing | -0.66 | Destabilizing | -2.082 | Destabilizing |
| *GLA*:c.335G>A:p.R112H | -1.726 | Destabilizing | -0.44 | Destabilizing | -1.883 | Destabilizing |
| *GLA*:c.409G>T; p.V137F | -1.328 | Destabilizing | -0.82 | Destabilizing | -1.47 | Destabilizing |
| *GLA*:c.413G>A:p.G138E | -2.6 | Destabilizing | -2.44 | Destabilizing | -2.842 | Destabilizing |
| *GLA*:c.494A>G:p.D165G | -0.712 | Destabilizing | -1.68 | Destabilizing | -0.981 | Destabilizing |
| *GLA*:c.548G>T:p.G183V | 0.655 | Stabilizing | -0.14 | Destabilizing | 1.043 | Stabilizing |
| *GLA*:c.627G>T:p.W209C | -1.853 | Destabilizing | -0.05 | Destabilizing | -1.449 | Destabilizing |
| *GLA*:c.657C>G p.I219M | -1.476 | Destabilizing | -0.52 | Destabilizing | -1.458 | Destabilizing |
| *GLA*:c.668G>A:p.C223Y | -1.419 | Destabilizing | -0.97 | Destabilizing | -1.565 | Destabilizing |
| *GLA*:c.680G>A:p.R227Q | -1.191 | Destabilizing | -1.95 | Destabilizing | -1.487 | Destabilizing |
| *GLA*:c.797A>G:p.D266G | -0.475 | Destabilizing | -0.41 | Destabilizing | -0.374 | Destabilizing |
| *GLA*:c.851T>C:p.M284T | -2.445 | Destabilizing | -3.37 | Destabilizing | -2.59 | Destabilizing |
| *GLA*:c.902G>A:p.R301Q | -0.125 | Destabilizing | -1.86 | Destabilizing | -0.49 | Destabilizing |
| *GLA*:c.1025G>A:p.R342Q | -1.393 | Destabilizing | -1.89 | Destabilizing | -1.675 | Destabilizing |
| *GLA*:c.1088G>A:p.R363H | -1.892 | Destabilizing | 0 | Destabilizing | -1.727 | Destabilizing |
