# Supplementary material for: Fabry disease in India: A multicenter study of the clinical and mutation spectrum in 54 patients
Source: JIMD Rep. 2020 Aug 15;56(1):82–94. doi: 10.1002/jmd2.12156 (PMC7653245; doi:10.1002/jmd2.12156)
Supplement: Supplementary file 8 — Supplementary Table S3 Protein 1R46 Pfam domain details [file JMD2-56-82-s008.docx]

**Supplementary Table 3**: Protein 1R46 Pfam domain details

|  | **Family** | **Description** | **Alignment** | | **E-value** |
| --- | --- | --- | --- | --- | --- |
|  |  |  | **Start** | **End** |  |
| 1 | Melibiase_2 | Alpha galactosidase A | 39 | 322 | 2.80E-162 |
| 2 | Melibiase_2_C | Alpha galactosidase A C-terminal beta | 325 | 411 | 4.50E-32 |
